# Reproducibility of deep inspiration breath‐hold technique for left‐side breast cancer with respiratory monitoring device, Abches

**DOI:** 10.1002/acm2.13529

**Published:** 2022-01-11

**Authors:** Masahide Saito, Daichi Kajihara, Hidekazu Suzuki, Takafumi Komiyama, Kan Marino, Shinichi Aoki, Koji Ueda, Naoki Sano, Hiroshi Onishi

**Affiliations:** ^1^ Department of Radiology University of Yamanashi Chuo City Yamanashi Japan

**Keywords:** DIBH, left‐side breast cancer, radiotherapy, reproducibility

## Abstract

**Purpose:**

This study aimed to evaluate the reproducibility of deep inspiration breath‐hold (DIBH) using a respiratory control device, Abches, in patients with left‐sided breast cancer.

**Material and methods:**

Abches comprises a main body, an indicator panel, and two fulcrums, one each on the chest and abdomen. Forty left side breast cancer patients treated with DIBH using abches were enrolled in this study. For all patients, CT images were taken three times to confirm the target position inside the body and to check the breath‐hold reproducibility. Three anatomical points on the nipple, sternum, and heart were selected as measurement points on CT images. After measuring the coordinates, breath‐hold reproducibility was defined as the mean of the absolute difference in the coordinates between the three CT images. The maximum differences were also investigated. In addition, the dice similarity coefficient (DSC) was calculated to examine the displacement of the heart volume in detail. Moreover, digitally reconstructed radiographs (DRRs) and linac graphs (LGs) were used to measure the positional accuracy of the chest and heart.

**Results:**

The reproducibility in all patients was within 0.75 mm for the nipple, 0.78 mm for the sternum, and 2.18 mm for the heart in each direction. Similarly, the maximum displacements for all patients were within 1.90 mm, 1.69 mm, and 4.75 mm, respectively, in each direction. For heart volume, the average DSC for all cases was 0.93 ± 0.01. The differences between the DRR and LG images were 1.70 ± 1.10 mm and 2.10 ± 1.60 mm, for the chest and heart, respectively.

**Conclusion:**

Our study showed that DIBH using Abches can be performed with good target reproducibility of less than 3 mm with proper breath‐hold practice, whereas the heartbeat‐derived reproducibility of the cardiac position is poor and needs to be monitored carefully during treatment simulation

## 1 INTRODUCTION

During radiotherapy for breast cancer, the exposure of the heart to ionizing radiation can increase the risk of ischemic heart disease.^1^ This is especially a problem during radiotherapy of the left breast, since the heart easily enters the irradiation field in these cases. To attenuate the risk, the deep inspiration breath‐hold (DIBH) technique, that has shown equivalent dosimetric effectiveness compared with that of free‐breathing,^2^ is frequently used for left‐sided breast cancer.

DIBH treatments have been performed with respiration‐control devices such as surface monitoring systems^3^ and reflective markers on the body surface.^4^ In addition, Onishi et al. developed a patient‐controlled respiratory device based on visual confirmation, in which fulcrums placed on the patient's abdomen and chest measured thoracoabdominal surface displacements.^5^ This device was subsequently commercialized under the name “Abches” (APEX Medical Inc., Tokyo, Japan), and has since been used in many facilities as a breath‐hold monitor. Recently, Abches was used for respiratory‐gated radiotherapy, and demonstrated good feasibility in terms of the delay time and beam characteristics, as per a previous report.^6^ As for its benefits in patients with left‐sided breast cancer, Lee et al. reported that the use of the device significantly reduced the radiation dose to the heart and left anterior descending artery (LAD), thereby potentially reducing cardiac risk.^7^ However, there have been no reports focusing on the reproducibility of DIBH using Abches in patients with left‐sided breast cancer. It is imperative to investigate the accuracy of breath‐hold while administering radiotherapy so as to curtail its ill‐effects. Therefore, this study aimed to evaluate the reproducibility of DIBH in patients with left‐sided breast cancer.

## 2 METHODS

### 2.1 Study design and selected patients

This was a retrospective study approved by our institutional review board. From January 2018 to June 2021, forty patients with left‐sided breast cancer, treated with DIBH using Abches, were enrolled in this study. Patient characteristics were follows: median age: 62 years (range: 35–77), prescription dose: 50 Gy/25 Fr in 16 cases, 42.56 Gy/16 Fr in 24 cases, irradiation technique: 3DCRT for 37 cases and IMRT(VMAT) for 3 cases.

### 2.2 Details and accuracy of the **Abches** device

**FIGURE 1:**
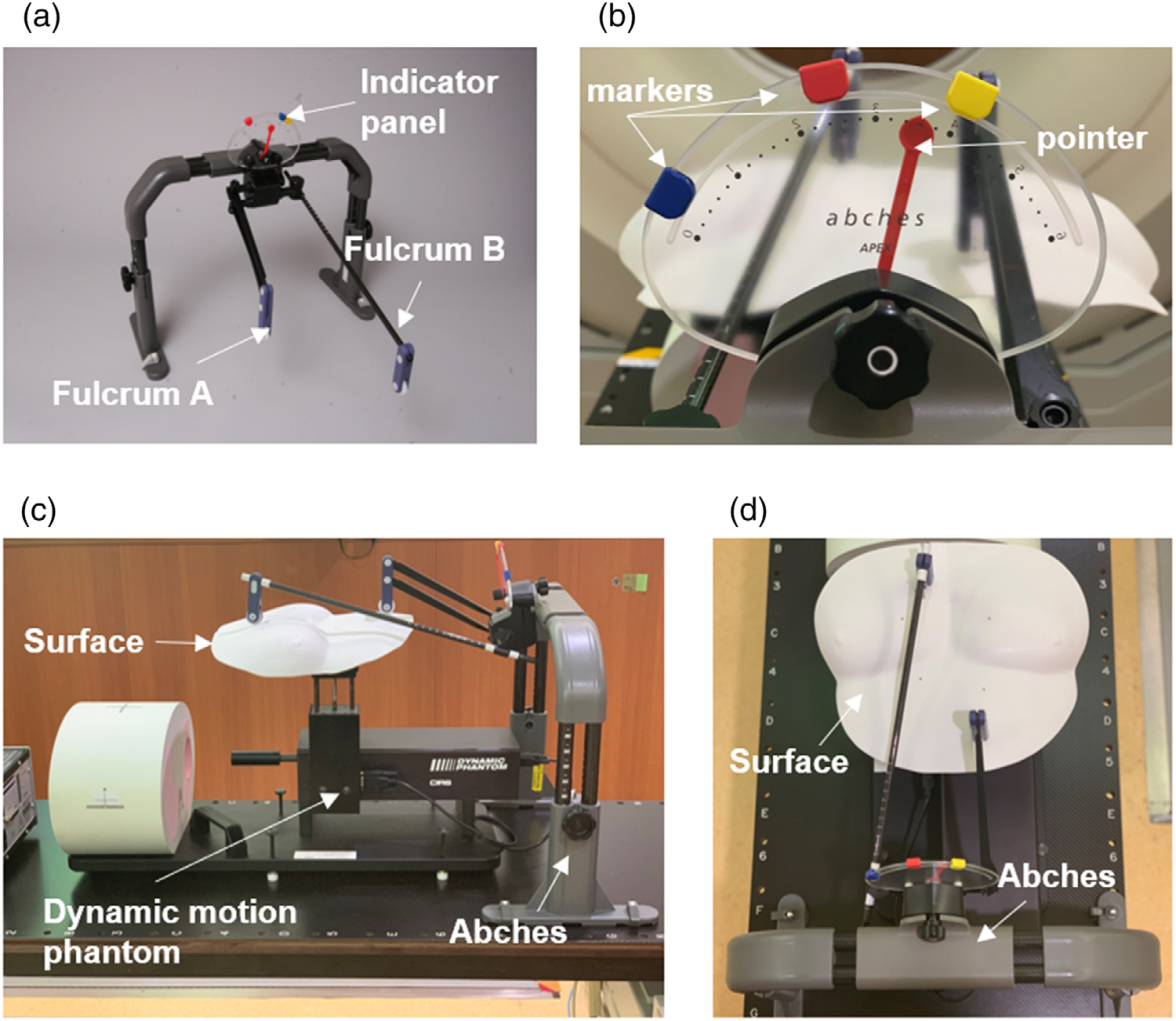
Overview of Abches device (a, b). Part (a) shows Abches comprising a main body, an indicator panel, and two fulcrums (A and B). Part (b) shows an enlarged view of the indicator panel which has a pointer and markers. Parts (c) and (d) show overview of the motion phantom setup to validate the accuracy of Abches.

The Abches device, as shown in Figure 1a, comprises a main body, an indicator panel, and two fulcrums. One fulcrum (fulcrum A) is placed on the patient's abdomen, while the other (fulcrum B) is placed on the chest, to measure thoracoabdominal surface displacements. Only one fulcrum (on the patient's chest) can also be used, especially for left‐sided breast cancer, but we routinely use two fulcrums at our institute. Figure 1b shows an enlarged view of the indicator panel. The pointer on the indicator panel moves in accordance with the fulcrums during respiration. The three markers can be set manually according to the respiratory range of the individual patient. In this figure, blue, red, and yellow indicate the expiration, inspiration, and deep inspiration positions, respectively. A mirror is usually attached to the patient's head, thereby allowing the patient to monitor the pointer position that correlates with his respiratory motion. The main unit can be used independently or can also be connected to a personal computer (PC) via a local area network (LAN) cable. Respiratory waveforms can be obtained as the output at sampling intervals of approximately 30 ms, and synchronization signals can be generated.^6^ Additional details regarding the Abches device are available in the previous report.^5^

In this study, a basic validation procedure was performed using phantom motion, to confirm the accuracy of Abches. Figure 1c and 1d shows an overview of the setup. A surface plate simulating a patient was placed on the surrogate of the phantom to simulate Abches use. Sin waveforms with amplitudes of ±2.5 mm, ±5.0 mm, and ±10 mm, at periods of 2.0 s, 4.0 s, and 8.0 s, were input to the dynamic phantom and compared with the output signal of the Abches. In addition, breath‐hold waveforms of 10 repetitions of a 10‐s breath‐hold were also investigated. The evaluation was performed using root mean square error (RMSE, mm) in Equation (1):

(1) $$\mathrm{RMSE}\;\left(\mathrm{mm}\right)=\sqrt{\frac{1}{n}\sum_{k=1}^{n}{\left(S_{\mathrm{ref},\;i}-S_{\mathrm{Abches},i}\right)}^{2},}$$

where *n* is the total number of sampling points for each respiration signal, $S_{\mathrm{ref},\;i}$ is the value of the amplitude (mm) from the reference input signal at the sampling point *i*, and $S_{\mathrm{Abches},i}$ is the value of the amplitude (mm) from the Abches output signal at sampling point *i*. The RMSE was calculated up to 40 s and 180 s for sin waveform and breath‐hold waveform, respectively. All signal analyses were performed using MATLAB 2019a (Natick, MA).

### 2.3 DIBH using **Abche**s

**FIGURE 2:**
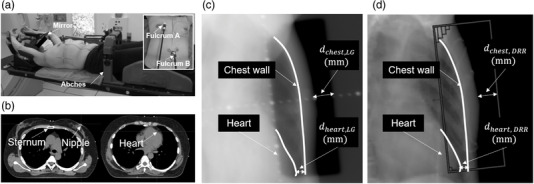
(a) The typical setup of Abches for a patient with left‐sided breast cancer. Fulcrum A is placed on the patient's abdomen and Fulcrum B is placed on the contralateral chest to measure the deep inspiration breath‐hold (DIBH) signal; (b) measurement points for the sternum, nipple, and heart; (c) a linac graph (LG) acquired before the treatment; (d) a digitally reconstructed radiograph (DRR) reconstructed from treatment planning CT image. Edge lines of the heart and chest wall are defined as white lines in each image

The setup of the Abches for a patient with left‐sided breast cancer is shown in Figure 2a. DIBH for left breast cancer was performed as per the following workflow: first, DIBH was practiced using the Abches before the simulation computed tomography (CT) scan. The technologist gave an audible cue and asked the patient to breathe as deeply as possible, to determine the appropriate breath‐hold range for each patient. Patients who were unable to hold their breath properly at this point were not eligible for treatment with DIBH. Next, CT images were taken three times to confirm the target position inside the body and to check the breath‐hold reproducibility.

In practice, we give the cue for DIBH, and perform CT imaging a few seconds later, after the patient is stabilized. The reproducibility of the relationship between the chest wall and the position of the heart is also checked. At the first treatment, a linac graph (LG) is acquired with the megavoltage (MV) beam just before irradiation. During the inter‐treatment period, CT imaging is not performed every time to minimize radiation exposure, but cone‐beam computed tomography (CBCT) is performed once a week for all patients to check the accuracy of the target position. During treatment in each fraction, we also carefully monitor the Abches’ respiratory waveform to ensure accuracy. When the waveform from the simulation is played, the timing of the breath‐hold must match that of simulation, making it difficult to send flexible breath‐hold cues. Therefore, we believe that it is desirable to check only the amplitude range between simulation and treatment during the actual operation.

### 2.4 Analysis

In order to investigate the reproducibility of DIBH in individual patients, we used three breath‐hold CT images acquired during treatment planning. All images were acquired using Aquilion LB (Canon Medical Systems, Otawara, Japan) at 120 kV and 20 mA with the following imaging parameters: field‐of‐view (FOV), 55 cm×55 cm; image size, 540 × 540 pixels; pixel size, 0.5 mm; and slice thickness, 2.0 mm.

Three anatomical points on the nipple, sternum, and heart were selected as the measurement points. After taking the first CT image, the second and third CT images were taken in succession without changing the patient's position. Next, we obtained the displacements of each point by comparing the points set in the first CT image with that in the second and third CT images. A typical measurement point is shown in Figure 2b. The measurement positions for the sternum and nipple were first set in the same slice of the first CT image as a reference. Then, the positions of each anatomical point set on the reference image were manually set on each CT image. Each point selection was performed by a radiology technician and a medical physicist. The respective displacements of the sternum and nipple were measured independently on different CT images. The heart was measured in positions that were easily identifiable for each patient, such as at the apex, ventricular septum, or near the LAD. The anterior–posterior (AP), left–right (LR), and superior–inferior (SI) coordinates of each measurement point were measured by using the MIM Maestro software (MIM Software Inc., Cleveland, OH).

After measuring the coordinates, the mean of the absolute difference in the coordinates between the three CT images was defined as breath‐hold reproducibility. The maximum differences were also investigated. Each index was calculated using Equation (2):

(2) $$\begin{array}{c} X_{1}=\left|x_{1}-x_{2}\right|,\;\;X_{2}=\left|x_{2}-x_{3}\right|,\;\;X_{3}=\left|x_{3}-x_{1}\right|\\ \mathrm{Reproducibility}=\bar{X},\;\;\mathrm{Maximum}\;\mathrm{displacement}=\max X \end{array},$$

where *x* is the coordinate in each CT image, and *X* is the absolute difference in coordinates between the CT images. This equation was adapted for all three axes.

In addition, the dice similarity coefficient (DSC) was calculated to examine the displacement of the heart in detail. The contours of the whole heart were delineated and examined in all CT images of 21 cases in which the heart was completely visible in the images. The DSC was calculated for each combination of the three CTs.

Furthermore, we evaluated the DIBH accuracy before the actual treatment. For 37 cases of 3DCRT, LG and digitally reconstructed radiograph (DRR) measurements were performed as shown in Figure 2c,d. The distance between the edge of the irradiation field and the chest wall at the center of the field was selected and defined as $d_{\mathrm{chest}}\;$. The position where the heart was the closest to the chest wall was selected within the irradiation field and defined as $d_{\mathrm{heart}}$. Next, scatter plots of the respective distances were made to investigate each correlation and to calculate the position error of the measurements between LG and DRR. Regarding the duration of breath‐hold, we conducted a ≥ 10‐s breath‐hold during treatment planning and LG imaging to reproduce the irradiation time as much as possible.

## 3 RESULTS

**FIGURE 3:**
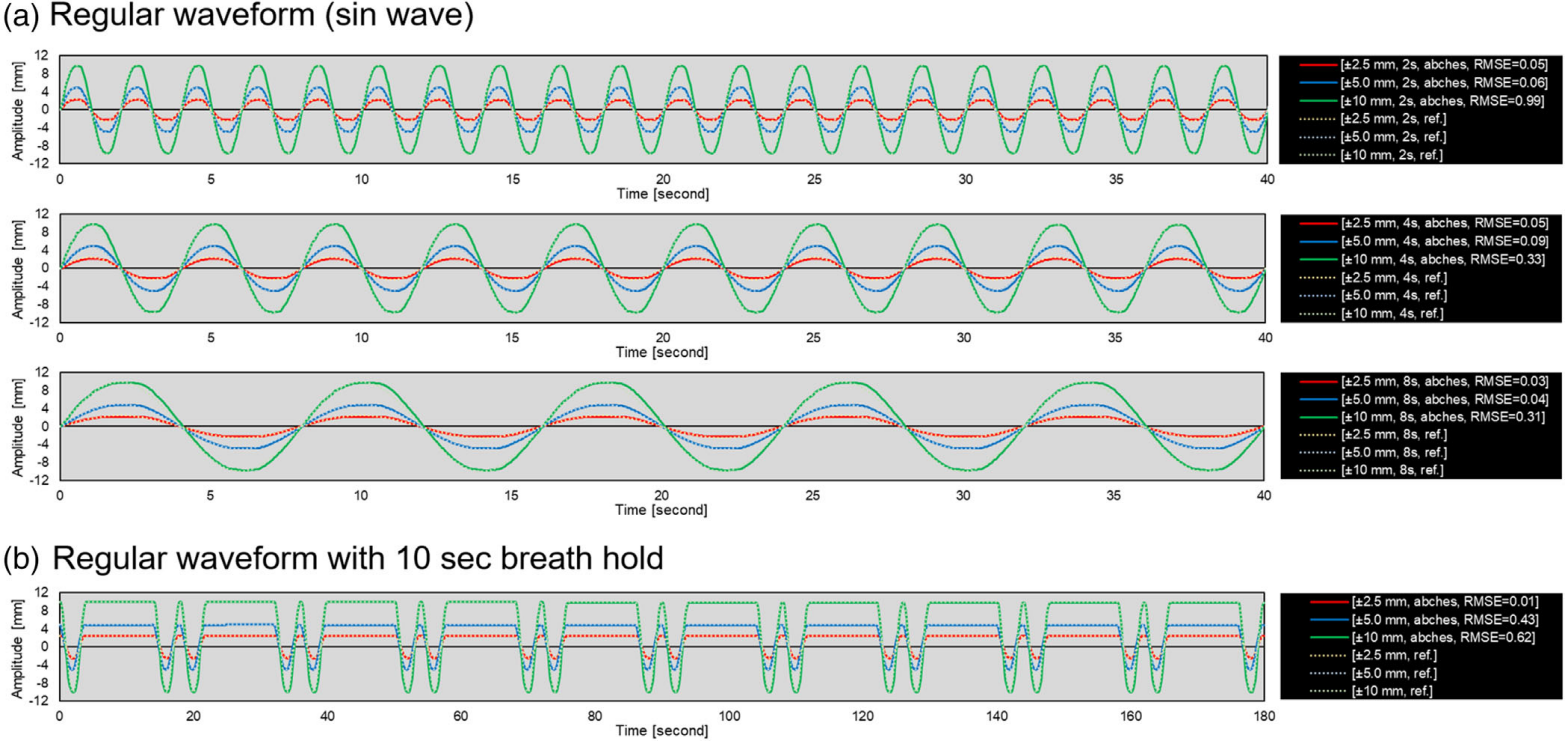
The results of the accuracy verification of Abches using the dynamic phantom. Part (a) shows the regular waveform, and part (b) shows the regular waveform with breath‐hold. The solid line shows the input signal from dynamic motion phantom (reference signal), and the dotted line shows the output signal from Abches. Each root mean square error (RMSE) was calculated by Equation (1)

Figure 3 shows the results of the experiment done to verify the accuracy of Abches using the dynamic phantom. The RMSE of the sin wave was 0.05 mm, 0.06 mm, and 0.99 mm for an amplitude of ±2.5 mm, ±5.0 mm, and ±10 mm, respectively, for a period of 2 s. The trend was the same for different cycles, with larger amplitudes resulting in larger RMSE. In the breath‐hold condition, the RMSE was 0.01 mm, 0.43 mm, and 0.62 mm for amplitudes of ±2.5 mm, ±5.0 mm, and ±10 mm, respectively.

The results of all the patients are summarized in Table 1. The reproducibility in all patients was within 0.75 mm for the nipple, 0.78 mm for the sternum, and 2.18 mm for the heart in each direction. Similarly, the maximum displacements for all patients were within 1.90 mm, 1.69 mm, and 4.75 mm, respectively, in each direction.

**TABLE 1:** The reproducibility and maximum displacement in forty patients with left‐sided breast cancer measured in CT images

		**Mean ± standard deviation (mm), *n* = 40**		
		SI	LR	AP
Nipple	Reproducibility	0.12 ± 0.36	0.75 ± 0.59	0.73 ± 0.66
	Maximum displacement	0.33 ± 0.85	1.59 ± 0.96	1.90 ± 1.32
Sternum	Reproducibility	0.26 ± 0.59	0.78 ± 0.78	0.72 ± 0.50
	Maximum displacement	0.45 ± 0.92	1.61 ± 1.42	1.69 ± 1.09
Heart	Reproducibility	2.18 ± 2.13	1.97 ± 1.85	1.44 ± 1.14
	Maximum displacement	4.75 ± 4.49	4.15 ± 2.91	3.08 ± 1.83

Abbreviations: AP, anterior–posterior; LR, left–right; SI, superior–inferior.

**FIGURE 4:**
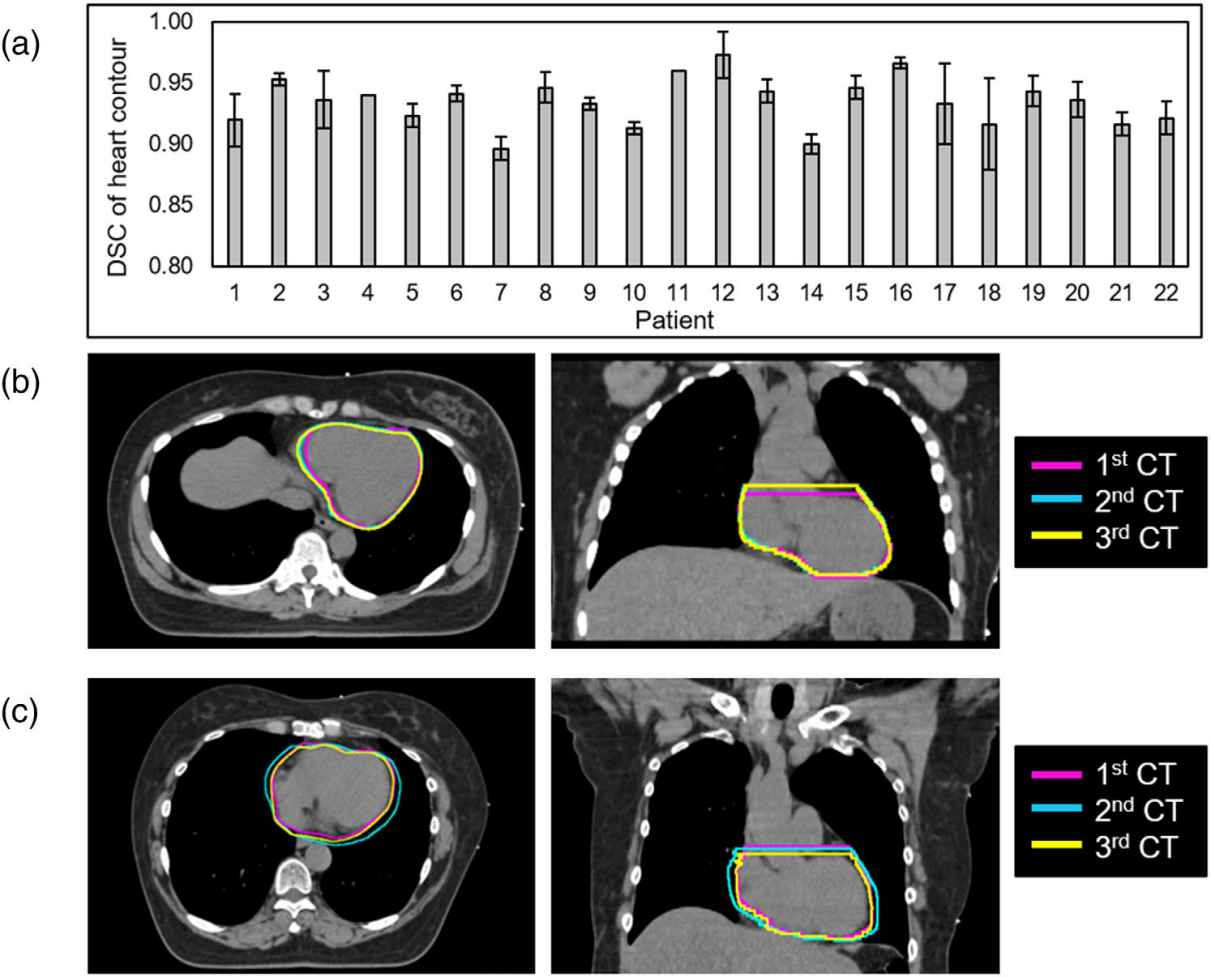
Average dice similarity coefficient (DSC) for all 21 cases in which the full range of the heart could be identified (a). The average DSC for all cases was 0.93 ± 0.01, indicating very good accuracy. Part (b) shows a case of good agreement of the heart position (patient 16) and part (c) shows a case of slight displacement of the heart position (patient 8).

Figure 4a shows the results of the DSC for all 21 cases in which the full range of the heart could be identified. The average DSC for all cases was 0.93 ± 0.01, indicating very good accuracy. Figure 4b shows a case of good agreement and Figure 4c shows a case of slight displacement of the cardiac position. In the latter case, the maximum local displacement was 7 mm, but the DSC was 0.90 ± 0.01, a good value.

**FIGURE 5:**
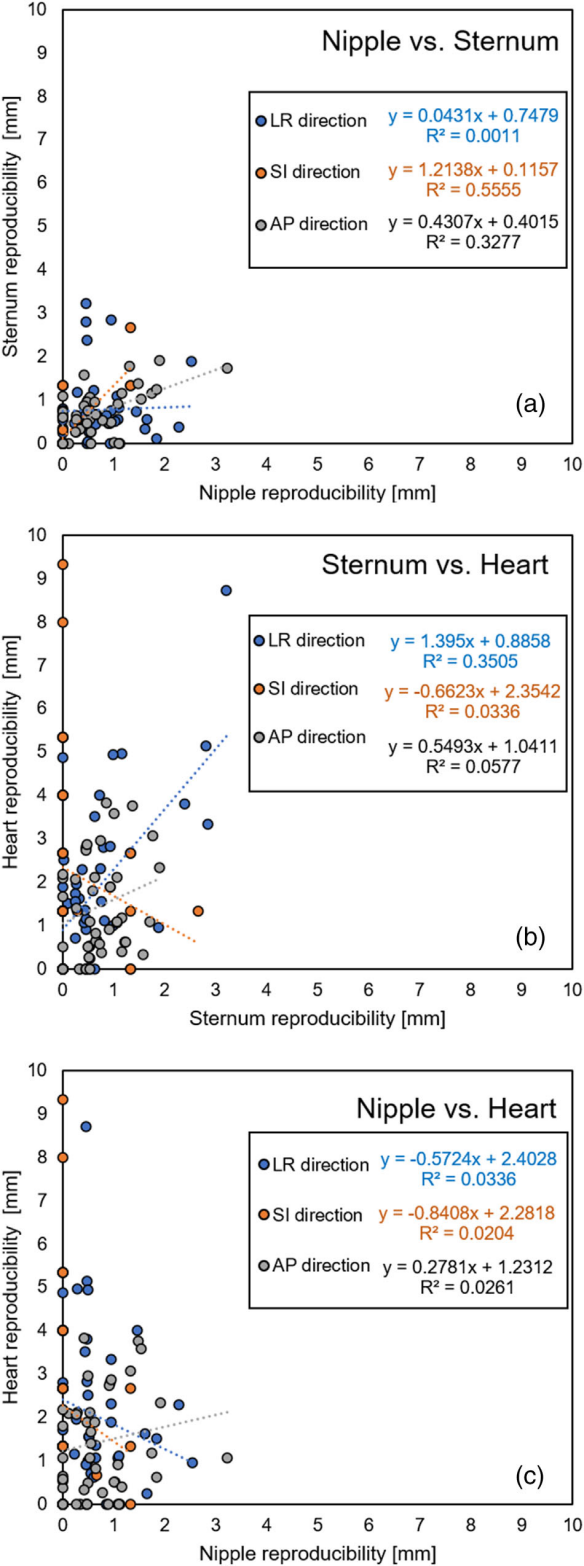
The scatter plots between the reproducibility of three sites (nipple, sternum, and heart) in the left–right (LR), superior–inferior (SI), and anterior–posterior (AP) directions are plotted for all patients. (a) Nipple vs. sternum, (b) sternum vs. heart, and (c) nipple vs. heart are shown. For each item, the approximate straight lines and *R* ^2^ values are shown

Figure 5 shows the scatter plots of the reproducibility (average value shown above) of each anatomical point in all 40 patients. There was no correlation between the reproducibility of each site. The reproducibility of the nipple and sternum was less than 4 mm, even in the largest patient. However, the reproducibility of the heart was more than 9 mm in some patients, despite the good reproducibility of the nipple and sternum.

**FIGURE 6:**
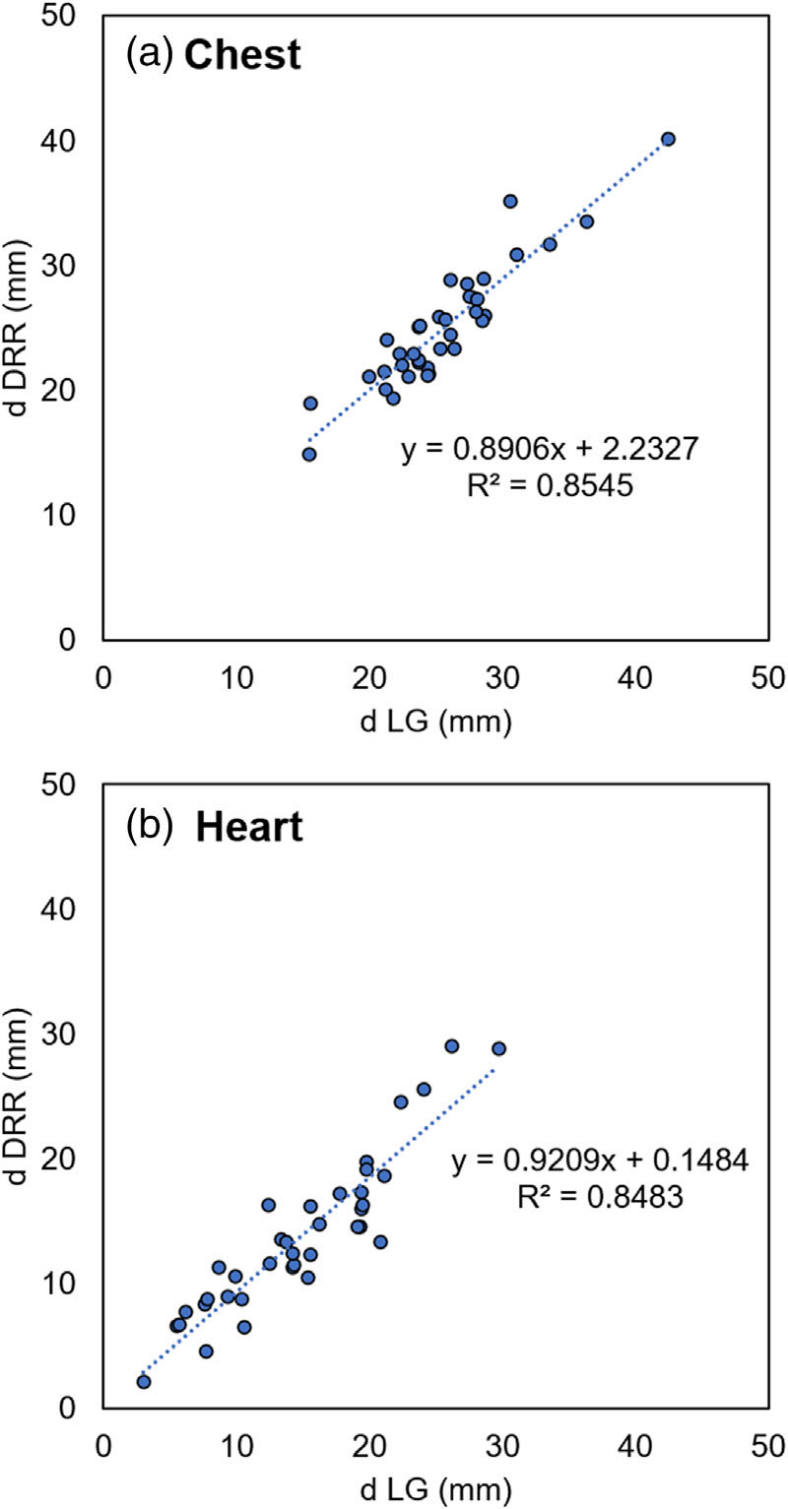
The scatter plots between the measurements of each distance in the digitally reconstructed radiograph (DRR) and the linac graph (LG), obtained before the treatment in 37 cases of 3DCRT(a) the result of $d_{\mathrm{chest}}$, which is defined as the distance between the edge of the irradiation field and the chest wall at the center of the field; (b) the result of $d_{\mathrm{heart}}$, which is defined as the position, where the heart was closest to the chest wall within the irradiation field

Figure 6 shows the measurement results of each distance in the DRR and LG, which were obtained before treatment in 37 cases of 3DCRT. Figure 6a shows the results for the distance between the edge of the irradiation field and the chest wall, while Figure 6b shows the results for the distance between the heart and the chest wall, and a high correlation was confirmed for each. Table 2 shows the mean values of the measurements for DRR, LG, and their differences in 37 cases of 3DCRT. The difference between DRR and LG images was 1.70 ± 1.10 mm and 2.10 ± 1.60 mm, for the chest and heart, respectively, indicating that the breath‐hold was well reproduced even before the treatment.

**TABLE 2:** The mean values of the DRR and LG measurements, and difference of both in 37 cases of 3DCRT

	Mean ± standard deviation (mm), *n* = 37		
	DRR	LG	Difference
$d_{\mathrm{chest}}$	25.7 ±5.00	25.1 ± 4.80	1.70 ± 1.10
$d_{\mathrm{heart}}$	14.8 ± 6.20	13.8 ± 6.20	2.10 ± 1.60

Abbreviations: DRR, digitally reconstructed radiograph; LG, linac graph.

## 4 DISCUSSION

The most important goal of DIBH in patients with left‐sided breast cancer is to reduce cardiac irradiation. Several dosimetric studies have examined DIBH for left‐sided breast cancer using Abches,^7^, ^8^, ^9^ and all of them have reported a reduction in cardiac irradiation with DIBH and Abches. However, the accuracy of breath‐hold is also important for successful treatment in actual clinical practice. To the best of our knowledge, no study has evaluated the accuracy (reproducibility) of DIBH for left‐sided breast cancer using Abches, thereby prompting us to investigate it. In order to see the reproducibility of true DIBH, we need data on the actual DIBH maintained during treatment. However, this was a retrospective study and we did not have such data. Therefore, in order to investigate the reproducibility of DIBH in individual patients, we used three breath‐hold CT images acquired at the time of treatment planning, and DRR and LG were used to evaluate the reproducibility before the treatment; since the duration of one DIBH was maintained at the same level as that during actual treatment, we believe that the results do not deviate significantly from those during treatment of implementation.

Since the chest wall is the target of therapy for left‐sided breast cancer, two anatomical points, the nipple and sternum, were set as measurement sites in this study. In addition, the position of the heart near the chest wall was also chosen since it may be included in the irradiation field. As for the measurement method, since the area of nipple is very small, and sternum is a rigid body, we assumed that only point measurements would be sufficient. The heart, on the other hand, is a non‐rigid body and movements are expected; therefore, we measured the volume in addition to the point measurements. During DIBH using Abches, the chest wall was limited to a movement of 3 mm, which was comparable to the results of sternal stability noted in a previous study using a surface monitor.^10^ For the left LAD motion, Jagsi et al. reported that the long‐term reproducibility was 3 mm in the AP, 7 mm in the SI, and 3 mm in the LR directions during DIBH.^11^ Our study showed that the movement of the heart was as much as 5 mm. Since it is impractical to control the heartbeat during radiotherapy, our results suggest that it is necessary to understand cardiac motion during breath‐hold, so as to accurately set the irradiation field.

This study had some limitations. This study did not evaluate the intra‐treatment DIBH reproducibility. We only evaluated the reproducibility before treatment using CT and LG images. Intra‐treatment evaluation warrants further study. Second, the SI direction was measured with a 2 mm slice CT, which may not be accurate. In our hospital, even if we obtain a reproducibility of 0 mm, we attempt to provide a margin of 2 mm in the SI direction. Third, no dosimetric data were included in this study since CT images were not acquired during free‐breathing, as this was a retrospective study. We routinely confirm the cardiac position in relation to the chest wall during DIBH under X‐ray fluoroscopy, which also helps reduce radiation exposure. If the relationship between dosimetric parameters and breath‐hold reproducibility is to be evaluated in the future, further studies are needed. Fourth, in the study using DSC, the heart was visible in 21 out of 40 cases, and that was not visible in the other cases. This was because we mainly focused on confirming the positional reproducibility of the chest wall and the left ventricular area in actual clinical practice. However, from the perspective of reducing radiation exposure, there was a certain number of cases where it was clinically acceptable even if the entire heart was not imaged. Finally, we examined Japanese people with a median age of 62 years. From the latest statistics, the median age of diagnosis of breast cancer for women in the United States is 63 years.^12^ However, some reports^13^, ^14^ from other countries have examined a younger age group, and the accuracy of DIBH using Abches could differ in terms of physical fitness and comprehension of the participants. Further research is needed on the adaptation of Abches for younger age groups.

## 5 CONCLUSION

This was the first report to evaluate the reproducibility of DIBH for left‐sided breast cancer using Abches. Our study showed that DIBH using Abches can be performed with good target reproducibility of less than 3 mm with proper breath‐hold practice, while the reproducibility of the cardiac position derived from the heartbeat is poor and needs to be monitored carefully during treatment simulation.

## CONFLICT OF INTEREST

The author declares that there is no conflict of interest that could be perceived as prejudicing the impartiality of the research reported.

## ETHICAL APPROVAL STATEMENT

This is retrospective study approved by University of Yamanashi Institutional Review Board (IRB) (Reference number: 2488).

## AUTHOR CONTRIBUTIONS

Study conception and design: Masahide Saito, Hidekazu Suzuki, Naoki Sano, and Hiroshi Onishi. Acquisition of data: Masahide Saito, Daichi Kajihara, Hidekazu Suzuki, Takafumi Komiyama, Kan Marino, Shinichi Aoki, and Koji Ueda. Analysis and interpretation of data: Masahide Saito, Daichi Kajihara, Hidekazu Suzuki, Naoki Sano, and Hiroshi Onishi. Drafting of manuscript: Masahide Saito and Hiroshi Onishi. Critical revision: Masahide Saito, Hidekazu Suzuki, Naoki Sano, and Hiroshi Onishi.

## Data Availability

The data that support the findings of this study are available on request from the corresponding author. The data are not publicly available due to privacy or ethical restrictions.
